# Disparities in influenza vaccination: Arab Americans in California

**DOI:** 10.1186/s12889-021-10476-7

**Published:** 2021-03-05

**Authors:** Rose-Marie Jungquist, Nadia N. Abuelezam

**Affiliations:** 1 School of Arts and Sciences, Boston College, Chestnut Hill, MA 02467 USA; 2 William F. Connell School of Nursing, Boston College, 140 Commonwealth Avenue, Chestnut Hill, MA 02467 USA

## Abstract

**Background:**

Influenza vaccination among minoritized groups remains below federal benchmarks in the United States (US). We used data from the 2004–2016 California Health Interview Surveys (CHIS) to characterize influenza vaccination patterns among Arab Americans in California.

**Methods:**

Influenza vaccination was self-reported by Arab American adults (*N* = 1163) and non-Hispanic Whites (NHW, *N* = 166,955). Differences in influenza vaccination prevalence and odds were compared using chi-squared tests and survey-weighted logistic regression, respectively.

**Results:**

Across all years, 30.3% of Arab Americans self-reported receiving an influenza vaccine (vs. 40.5% for NHW, *p* < 0.05). After sequential adjustment by sociodemographic, health behavior, and acculturation variables no differences in odds of self-reported influenza vaccination were observed between Arab Americans and NHW (odds ratio: 1.02, 95% confidence interval: 0.76–1.38). Male and unemployed Arab Americans had higher odds of reporting influenza vaccination than female and employed Arab Americans.

**Conclusions:**

Future work should consider specific barriers to influenza vaccination in Arab American communities.

## Introduction

Influenza is a contagious disease affecting millions of individuals in the United States (US) each year, resulting in illness, flu-related complications, and thousands of deaths [1, 2]. An annual influenza vaccine that is effective at reducing morbidity and mortality is available to all individuals ages 6 months and older [1]. Yet, influenza vaccination remains well below federally established benchmarks each year in minoritized populations, preventing optimal coverage [3]. Previous studies have considered whether disparities in vaccine uptake exist across ethnic/racial groups and in comparison to non-Hispanic whites (NHW) in the US [4–10]. African-American/Black, Hispanic, and Asian minorities have been found to receive disproportionately fewer influenza vaccines [4–10]. Arab Americans, a historically understudied minoritized group of approximately 3.7 million individuals in the US, have been largely excluded from consideration in vaccination studies [11, 12].

To our knowledge, only two previous studies have examined influenza vaccine uptake among Arab Americans [13, 14]. Based on 12 years of data obtained from the National Health Interview Survey, foreign-born Arab Americans were less likely to receive the influenza vaccine than both foreign-born and US-born Whites [13, 14]. However, both studies only considered data up until 2011, were solely focused on foreign-born Arab Americans, and used fairly small sample sizes (238 males, and 205 females). The present study used data from the California Health Interview Survey (CHIS) to compare influenza vaccination among Arab Americans from 2004 to 2016 in California to NHW and assessed predictors for influenza vaccination among Arab Americans.

## Methods

### Survey

Data were analyzed from the CHIS, the largest state health survey conducted in the US. The CHIS utilizes a dual-frame landline and cell phone random-digit-dialing sample of California’s non-institutionalized population. From 2001 to 2009 the CHIS survey was conducted every 2 years, and from 2009 to 2016 the survey was conducted annually. For this study, we analyzed data from the 2004–2016 CHIS survey cycles. More information on the CHIS can be found in previous work [15, 16].

### Study population

As previously described, Arab Americans were identified using their responses to three questions on the CHIS Adult Questionnaire related to place of birth, language spoken at home, and parents’ place of birth [15, 16]. If one of 22 Arab League countries were indicated for participant’s or parent’s place of birth, then respondents were coded as Arab American. If the respondent indicated that Arabic language was spoken at home, then they were coded as Arab American.

NHW respondents were isolated by their responses to the following questions: “Are you Latino or Hispanic?” and “Please tell me which one or more of the following you would use to describe yourself?” If the respondent indicated “No” for Hispanic or Latino origin, selected “White” for their racial identification, and was determined to not be of Arab descent, then they were coded as NHW.

### Outcome variable

The outcome variable evaluated in this study was influenza vaccination within the past year. Participants were asked if they had received a flu shot within the past 12 months (yes/no). Influenza vaccination was assessed for each survey cycle between 2004 and 2016.

### Covariates

Demographic variables, assessed for all survey years, include the following: sex (female vs. male), age (18–44 vs. 45+), marital status (married vs. never married), and length of time living in the US (born in the US vs. 0–14 years vs. greater than 15 years). Socioeconomic variables examined for all survey years include unemployment status (employed vs. unemployed), educational attainment (high school or less vs. some college vs. graduate degree), living below the federal poverty level (< 200% vs. ≥200%), and health insurance status (uninsured vs. insured). Health variables included smoking status (current vs. not current smoker) and obesity (overweight/obese vs. not overweight/obese). Covariates were chosen based on their availability in the dataset and their potential influence on influenza vaccination.

### Statistical analysis

Data were analyzed using SAS version 9.4 (SAS Institute, Cary, NC, 2013). Jackknife survey weights, provided by CHIS and implemented using Proc Surveylogistic, were used to account for non-response, compute standard errors and obtain weighted prevalence estimates based on the total population in California. Univariate comparisons between Arab Americans and NHW were made using chi-squared statistics. Vaccination prevalence was calculated for the 2015–2016 CHIS survey years in sensitivity analyses to compare to CDC data on influenza vaccination among minoritized groups during that time period. Survey-weighted logistic regression models were run with influenza vaccination as the main outcome. In the main model (Model 1), Arab ethnicity was used as the primary exposure. A series of sequential models were run. Model 2 was adjusted for age, sex, and marital status. Model 3 was additionally adjusted for education, employment, and federal poverty level. Model 4 was additionally adjusted for smoking and overweight/obesity. And Model 5 was additionally adjusted for number of years lived in the US. This sequential adjustment allowed us to examine the impact of sociodemographic, health, and acculturation related variables. A survey-weighted logistic regression model was run in the restricted sample of only Arab Americans to assess the predictors for influenza vaccination.

## Results

### Sample description

Our sample consisted of 1163 Arab Americans (weighted frequency: 121,294) and 166,955 NHW (weighted frequency: 9,854,763). Arab Americans in this sample were younger and had more males represented than in NHW (Table 1). There were more Arab American immigrants (born outside the US) than NHW immigrants in the sample.

**Table 1:** Comparison of Arab American and non-Hispanic White populations in the California Health Interview Survey data from 2004 to 2016 with data on influenza vaccination uptake

	Arab (***N*** = 1163)	Non-Hispanic White (NHW, ***N*** = 166,955)	
**Age**			*p* < 0.01
18–44	512 (65.8)	31,607 (36.6)	
45+	651 (34.2)	135,348 (63.4)	
**Sex at birth**			*p* = 0.044
Male	565 (55.0)	68,420 (48.9)	
Female	598 (45.0)	98,535 (51.1)	
**Year of Survey**			
2003–2007	509 (43.8)	74,501 (44.6)	
2008–2012	403 (34.6)	56,633 (33.9)	
2012–2017	251 (21.6)	35,821 (21.5)	
**Marital Status**			*p* < 0.01
Married	663 (51.7)	84,885 (57.0)	
Widowed/Separated/Living with Partner	241 (12.2)	61,036 (25.1)	
Never married	259 (36.1)	21,034 (17.9)	
**Education**			< 0.01
High school or less	269 (23.9)	41,978 (28.7)	
Some college	254 (23.0)	49,273 (27.5)	
College degree	390 (36.7)	44,102 (26.6)	
Graduate degree	247 (13.4)	31,528 (17.2)	
**Employment Status**			
Employed	667 (65.8)	84,878 (61.9)	
Unemployed	496 (34.2)	82,077 (38.1)	
**Poverty Level**			*p* < 0.01
< 200% of FPL	365 (36.1)	32,149 (17.7)	
> 200FPL	798 (63.9)	134,806 (82.3)	
**Smoking status**			*p* = 0.02
Current smoker	162 (19.3)	20,501 (14.2)	
Not current smoker	1001 (80.7)	146,454 (85.8)	
**Overweight or obese**			
Overweight or obese	704 (56.8)	94,669 (56.3)	
Not overweight or obese	459 (43.2)	72,286 (43.7)	
**Years Living in the US**			*p* < 0.01
Born in US	405 (37.3)	154,239 (91.3)	
0–14 years in US	209 (23.6)	1880 (2.1)	
15+ years in US	549 (39.1)	10,836 (6.6)	
**Health Insurance**			*p* < 0.01
Insured	761 (75.6)	88,330 (85.3)	
Uninsured	150 (24.4)	12,982 (14.7)	

Across all years, 30.3% of Arab Americans and 40.5% of NHW reported receiving an influenza vaccine. When restricting to the 2015–2016 CHIS survey year, prevalence of influenza vaccination among Arab Americans was 34.4%.

### Models

In unadjusted models, Arab Americans had reduced odds of reporting an influenza vaccination compared to NHW (OR: 0.64, 95% CI: 0.49, 0.83) (Table 2). After sequential adjustment for sociodemographic factors, health behaviors, and years living in the US, the odds of influenza vaccination did not differ for Arab Americans and NHW (Table 2).

**Table 2:** Logistic regression models comparing self-reported influenza vaccination uptake among Arab American adults in California to non-Hispanic Whites

Odds ratio (95% CI)	Model 1: Unadjusted	Model 2: Adjusted for age and marital status	Model 3: Model 2 + education, employment, and FPL	Model 4: Model 3 + smoking + overweight/obese	Model 5: Model 4 + years in the US
Arab American vs. NHW	0.64 (0.49, 0.83)	0.90 (0.67, 1.21)	0.89 (0.67, 1.19)	0.90 (0.67, 1.20)	1.02 (0.76, 1.38)
Female vs. male		1.21 (1.17, 1.26)	1.13 (1.09, 1.18)	1.14 (1.09, 1.19)	1.14 (1.09, 1.19)
45+ vs. 18–44		2.83 (2.68, 2.99)	2.35 (2.22, 2.48)	2.27 (2.14, 2.41)	2.23 (2.10, 2.36)
Never married vs. married		0.69 (0.65, 0.74)	0.72 (0.67, 0.78)	0.74 (0.69, 0.80)	0.73 (0.68, 0.79)
Other vs. married		0.90 (0.86, 0.95)	0.92 (0.87, 0.97)	0.96 (0.91, 1.01)	0.95 (0.90, 1.01)
College vs. graduate school			0.70 (0.66, 0.74)	0.71 (0.67, 0.75)	0.70 (0.66, 0.74)
High school or less vs. graduate school			0.56 (0.53, 0.60)	0.59 (0.56, 0.63)	0.58 (0.55, 0.62)
Some college vs. graduate school			0.59 (0.55, 0.62)	0.61 (0.57, 0.64)	0.60 (0.56, 0.63)
unemployed vs. employed			2.25 (2.17, 2.34)	2.23 (2.15, 2.32)	2.24 (2.15, 2.33)
< 200% FPL vs. > 200% FPL			0.79 (0.74, 0.85)	0.82 (0.77, 0.88)	0.83 (0.77, 0.89)
Current smoker vs. not current smoker				0.61 (0.57, 0.65)	0.61 (0.57, 0.65)
Overweight obese vs not				1.11 (1.06, 1.15)	1.10 (1.05, 1.14)
0–15 years vs. born in US					0.60 (0.49, 0.74)
15 + years vs. born in US					0.88 (0.82, 0.94)

Abbreviations- *NHW* Non-Hispanic White, *FPL* federal poverty level

Among Arab Americans, females had lower odds of influenza vaccination than males (OR: 0.57, 95% CI: 0.35, 0.94) (Table 3). A significant predictor of influenza vaccination among Arab Americans was being unemployed (OR: 1.86, 95% CI: 1.06, 3.28), even after adjustment for age (Table 3).

**Table 3:** Logistic regression model results examining predictors of influenza vaccination uptake among Arab Americans in California between 2004 and 2016

	Odds Ratio (95% CI)
Female vs. male	0.57 (0.35, 0.94)
45+ vs. 18–44	0.99 (0.55, 1.79)
Never married vs. married	0.87 (0.44, 1.74)
Other vs. married	1.44 (0.72, 2.88)
College vs. graduate school	0.77 (0.39, 1.54)
High school or less vs. graduate school	0.56 (0.28, 1.15)
Some college vs. graduate school	0.63 (0.33, 1.20)
Unemployed vs. employed	1.86 (1.06, 3.28)
< 200% FPL vs. > 200% FPL	1.00 (0.54, 1.86)
Current smoker vs. not current smoker	0.65 (0.27, 1.55)
Overweight obese vs not	1.03 (0.57, 1.86)
0–15 years vs. born in US	0.45 (0.19, 1.15)
15 + years vs. born in US	0.96 (0.53, 1.75)

Abbreviation- *FPL* federal poverty level

## Discussion

This study aimed to estimate the prevalence of influenza vaccination among Arab Americans in California and to compare the odds of vaccination to NHW. In unadjusted analyses, odds of influenza vaccination were significantly lower among Arab Americans than NHW. However, after adjustment for sociodemographic, health behavior, and acculturation information, no significant difference in odds of vaccination was observed between Arab Americans and NHW. In order to reduce cases of acute respiratory illness and to mitigate compounding stress on the health care system amid the COVID-19 pandemic, it is especially important to increase uptake of the influenza vaccine [17]. The prospect of a vaccine to mitigate the effects of the COVID-19 pandemic also requires a better understanding of vaccination behavior in immigrant groups.

The prevalence of influenza vaccination among Arab Americans in California during the 2015–2016 survey year was 34.4%. National data from the CDC shows that among all adults aged 18 years or older, influenza vaccination prevalence was 41.7% during the same time period. Flu vaccination prevalence among racial and ethnic subgroups varied with 36.6% of non-Hispanic Blacks, 34.4% of Hispanics, 44.0% of Asians, 42.9% of American Indian and Alaskan Natives, and 36.4% of Other or multiple race having reported receiving an influenza vaccine between 2015 and 2016 [18]. This suggests that Arab Americans (along with Hispanic Americans) are not receiving influenza vaccinations at the same rates as other racial and ethnic groups.

In 2011–2012, Latinos in California had the lowest unadjusted flu vaccination rates amongst NHW and other racial and ethnic groups [19]. However, after adjustment for sociodemographic and access-to-care variables, no difference was observed between Latinos and NHW, suggesting that this minoritized group is facing significant barriers to influenza vaccine access [19].

When compared to NHW, Arab Americans in our study were significantly more likely to live below the federal poverty line and to be uninsured. Given that higher income is considered a facilitator of influenza vaccination [20], and that adults without health insurance are unlikely to get vaccinated [20], we propose that income and insurance status may be two significant barriers to vaccination for Arab Americans. Other potential barriers to vaccination that Arab Americans may be facing are discrimination and stigmatization [21]. Persons who report discrimination are shown to be less likely to receive preventive services, including flu vaccines [22]. Additionally, delivery of care may be affected by unconscious provider bias, such that minoritized patients are less likely to be vaccinated than NHW patients [23].

Among racial and ethnic groups, numerous studies have indicated the importance of knowledge and beliefs about influenza and the vaccine as determinants of influenza vaccination [20, 23–28]. A nationally representative sample of US adults showed that minoritized groups’ flu-related beliefs were most strongly associated with vaccine uptake, in comparison to other sociodemographic factors [25]. It is possible that the disparity in vaccine uptake observed in our study is partially attributed to differences in perceived risk of the flu and efficacy of the vaccine. However, given that the CHIS does not collect data on health-related beliefs, we were unable to explore this theory further.

Previous studies on influenza vaccination prevalence among foreign-born Arab Americans in National Health Interview Surveys found that Arab Americans were less likely to report influenza vaccination than NHW [13, 14]. Dallo and Kindratt reported an odds ratio of 0.48 for vaccination among foreign-born Arab American women and an odds ratio of 0.42 for vaccination among foreign born Arab American males in unadjusted models when compared to NHW [13, 14]. These vaccination odds were lower than those observed in our study; it is notable that these studies did not include US-born Arab Americans while ours did. In our Arab-only model, being born in the US increased the likelihood of influenza vaccination, although the results were not statistically significant. Our results support the notion that health behaviors differ with each immigrant generation [16]. Influenza vaccination remains uncommon in the MENA region, in part due to low vaccine efficacy, as well as misbeliefs and fear concerning the vaccine [29, 30]. Vaccination rates may increase with each Arab American generation due to separation from these health beliefs.

Among Arab Americans, unemployed adults had significantly greater odds of reporting a flu vaccination. Similarly, a study of influenza vaccination in minoritized populations reported that unemployed elderly individuals were more likely to be vaccinated [31]. Across minoritized and NHW populations, the elderly (≥65) are routinely found to have the highest coverage of influenza vaccine [32]. Given that a significant proportion of the Arab American elderly population in our study reporteded being unemployed, we believe this explains the observed correlation between influenza vaccination and unemployment.

### Limitations and strengths of this study

There are some limitations to our study. First, a representative sample of Arab Americans may not have been captured by random digit dialing for recruitment. Arab Americans were notably younger than NHW in this sample. It is possible that restricting the survey to English speaking individuals created an age bias among Arab Americans. Second, all outcomes in the survey were self-reported by the participants and were not verified with medical records. Third, the group of Arab Americans isolated by the CHIS is heterogeneous, as individuals from a wide breadth of diverse Arabic speaking countries are included, which may mask differential vaccination patterns for subgroups of this population. Despite these limitations, there are a number of strengths of this work. Most notable is the utilization of a large, population-representative sample of first, second, and third generation Arab Americans in the state with the largest Arab American population in the US.

## Conclusions

Our study provides one of the first looks at influenza vaccination amongst Arab Americans in California. The results of this study emphasize the importance of disaggregating Arab Americans from NHW in future studies of vaccination prevalence. Future work should allow for Arab American ethnicity to be self-identified in order to capture the health outcomes and health needs of this minoritized population. Given that vaccination behaviors among Arab Americans are understudied, concerted research in this area is necessary to identify specific barriers to influenza vaccination in Arab American communities. The development of culturally and linguistically specific interventions is imperative. Our results indicate that a dedicated effort will have to be made to vaccinate Arab Americans in California.

## Data Availability

The data underlying this article is publically available and is housed by the UCLA Center for Health Policy Research. Data can be accessed upon request to the UCLA Center for Health Policy Research.
